# Enhanced Mechanical Properties of Mn- and Fe-Doped Na_0.5_Bi_0.5_TiO_3_ Ceramics

**DOI:** 10.3390/ma17225645

**Published:** 2024-11-19

**Authors:** Jan Suchanicz, Marcin Wąs, Kamila Kluczewska-Chmielarz, Grzegorz Jagło, Dagmara Brzezińska, Roman Rosiek, Grzegorz Stachowski, Mariusz Sokolowski

**Affiliations:** 1 Department of Mechanical Engineering and Agrophysics, University of Agriculture in Krakow, Balicka 120, 31-120 Krakow, Poland; 2 Department of Bioprocess Engineering, Power Engineering and Automation, University of Agriculture in Krakow, Balicka 116, 31-120 Krakow, Poland; 3 Institute of Technical Sciences, University of the National Education Commission, Podchorazych 2, 30-084 Krakow, Poland; kamila.kluczewska-chmielarz@uken.krakow.pl (K.K.-C.); grzegorz.jaglo@uken.krakow.pl (G.J.); roman.rosiek@uken.krakow.pl (R.R.); 4 Institute of Materials Engineering, Faculty of Science and Technology, University of Silesia in Katowice, 75 Pułku Piechoty 1a, 41-500 Chorzow, Poland; dagmara.brzezinska@us.edu.pl; 5 Astronomical Observatory, Jagiellonian University, Orla 171, 30-244 Krakow, Poland; grzegorz.stachowski@uj.edu.pl; 6 Faculty of Computer Science, Electronics and Telecommunications, AGH University of Science & Technology, 30-059 Krakow, Poland; sokolows@agh.edu.pl

**Keywords:** Mn- and Fe-doped sodium bismuth titanate Na_0.5_Bi_0.5_TiO_3_, mechanical properties, stress–strain behavior, fracture toughness, hardness, bending strength

## Abstract

The mechanical properties of Mn- and Fe-doped Na_0.5_Bi_0.5_TiO_3_ ceramics in unpoled and poled states were examined and analyzed for the first time through measurements of Young’s modulus, the elastic modulus, Poisson’s number, compressibility modulus K, hardness, fracture toughness and bending strength on one hand and by stress–strain measurements on the other hand. It was found that both the introduction of Fe and Mn ions into Na_0.5_Bi_0.5_TiO_3_ and E-poling lead to improvements in their mechanical properties. The additives also cause improvement of the piezoelectric properties. The stress–strain curves revealed a changing mechanical response with the Mn and Fe doping of the NBT. With the doping, there was a decrease in coercive stress, which enhanced the remnant strain. In contrast, the E-poling led to an increase in the coercive stress, which reduced the remnant strain. Induced internal stresses associated with non-180° domain switching were determined. It was found that the investigated materials displayed significant ferroelastic deformation and large remnant polarization even under external stress of 180–250 MPa. Modification of NBT by Mn and Fe ions and E-poling were found to be effective ways of improving actuator performance and controlling operating stresses in order to minimize irreversible fatigue damage. The results suggest that the investigated materials could replace PZT ceramics in actuator applications where high blocking stress is required.

## 1. Introduction

Lead-free ferroelectric/piezoelectric ceramics have been extensively studied in response to legislation introduced by European Union RoHS/WEEE regulations. One promising material is Na_0.5_Bi_0.5_TiO_3_ (NBT). NBT exhibits high remnant polarization (P_r_ = 30 μC/cm^2^) and relatively high depolarization temperature (T_d_ = 170–200 °C). It is a multiaxial ferroelectric material in which ferroelastic properties exist [1]. Ferroelastics are the mechanical analogues of ferroelectrics, with spontaneous strain replacing spontaneous polarization, uniaxial stress replacing the electric field and mechanical domain structure (twins) and stress–strain hysteresis (ferroelastic strain hysteresis) replacing ferroelectric domain structure and electric hysteresis, respectively. In multiaxial ferroelectrics, ferroelectric and ferroelastic domains coincide, and they can be switched/moved both by uniaxial pressure and by an electric field.

In many applications, ferroelectric ceramics are subjected to electric fields and mechanical stress, under which their properties are not yet well-examined or -understood. Knowledge of these properties, particularly their mechanical behavior in the unpoled/poled states, is relevant from both the application and scientific points of view. PZT-based ceramics are currently the most important piezoceramics in practical applications. However, due to increasingly stringent legal restrictions on the use of toxic lead in electric and electronic equipment, research and development of lead-free alternatives are being conducted. Under certain conditions, the electromechanical properties of some compositions now match or even surpass commercially available lead-containing materials over a wide temperature range, making them potentially attractive for non-resonant displacement applications [2,3]. Ferroelastic toughening has been extensively demonstrated for PZT as a function of temperature [4,5], grain size [6] and poling state [5,6]. However, there is a lack of fracture studies in lead-free ferroelectrics, in particular those that couple results with macroscopic mechanical properties [2,3,7,8,9,10,11]. In addition, there is currently very little understanding of the mechanical reliability of lead-free ferroelectrics [3,4]. It has been demonstrated that mechanical strength can be improved by controlling the quenching rate [12], and the enhancement of mechanical properties through the introduction of arrays of ordered dislocations has also been proposed [13]. PZT-based ceramics are also known to have low fracture toughness, which makes them susceptible to fractures. It is therefore necessary to examine the fracture resistance of lead-free materials that might possibly replace existing PZT-based ceramics. A few papers have reported the properties of Mn- and Fe-doped NBT [14,15,16,17,18,19]; however, most of them are limited to unpoled states. There are also no studies on the mechanical properties (i.e., hardness, elastic modules, fracture toughness) of these materials. In this work, the influence of the addition of Mn and Fe ions into NBT ceramics on their mechanical properties in both unpoled and poled states were examined and analyzed. The results were contrasted to stress–strain behavior.

## 2. Experimental Procedure

**Figure 1:**
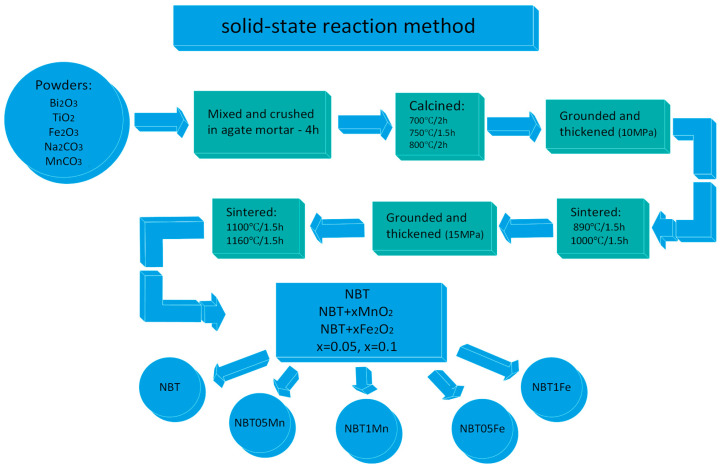
Schematic diagram showing technological conditions for production of the samples under investigation.

NBT + xMnO_2_ and NBT + xFe_2_O_3_, x = 0.05 and 0.1 (NBT05Mn, NBT1Mn and NBT05Fe, NBT1Fe, respectively), were prepared using the solid-state reaction method on the oxide and carbonate powders Bi_2_O_3_ (99.95%, POCH), TiO_2_ (99.95%, POCH), Fe_2_O_3_ (99.95%, POCH), Na_2_CO_3_ (99.95%, POCH) and MnCO_3_ (99.95%, POCH). After weighing, the starting reactants were mixed and crushed in an agate mortar for 4 h. The obtained reagent was calcined under the following conditions: 700 °C/2 h, 750 °C/1.5 h and 800 °C/2 h. After calcination, the material was ground and thickened by pressing under a pressure of 10 MPa and sintered under the following conditions: 890 °C/1.5 h and 1000 °C/1.5 h. Then the material was ground again and pressed under a pressure of 15 MPa. The obtained mixtures were sintered at temperatures of 1100 °C/1.5 h and 1160 °C/1.5 h (Figure 1).

The mechanical properties (Young’s modulus (E), elastic modulus (G), Poisson’s number (μ) and compressibility modulus (K)) were studied by an ultrasonic pulse echo technique, as has been reported elsewhere [20,21]. For the stress–strain measurements, cylindrical samples with nominal dimensions of 6 mm × 2 mm × 2 mm were prepared. The samples were preloaded to 5 MPa and subjected to compressive stress of up to maximum loads of 550 MPa, parallel to the poling direction, with a loading rate of 5 MPas^−1^. The well-polished ceramics were subjected to the microhardness tester (Vickers hardness, H_V_). The indentation load used was 1.96 N, which was applied for a 15 s holding time. Bending strength was measured by the three-point method.

## 3. Results and Discussion

**Figure 2:**
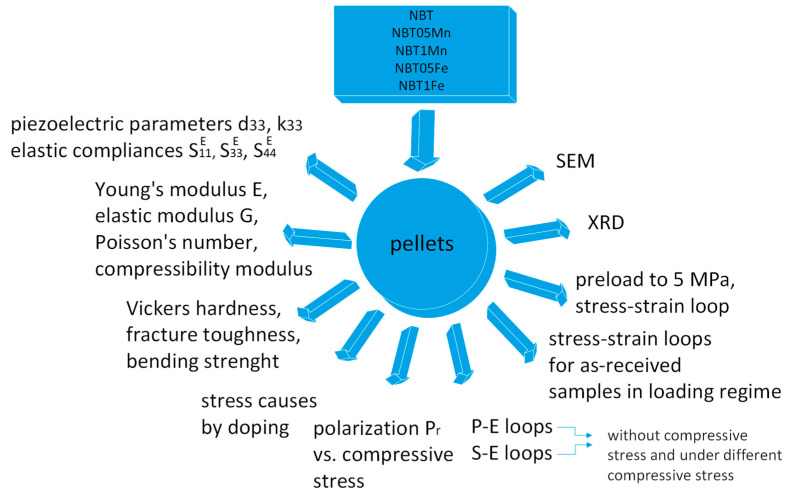
Schematic illustration of performed experiments.

Figure 1 and Figure 2 present a schematic diagram showing the technological conditions for the production of the samples under investigation and a schematic illustration of the performed experiments, respectively.

**Figure 3:**
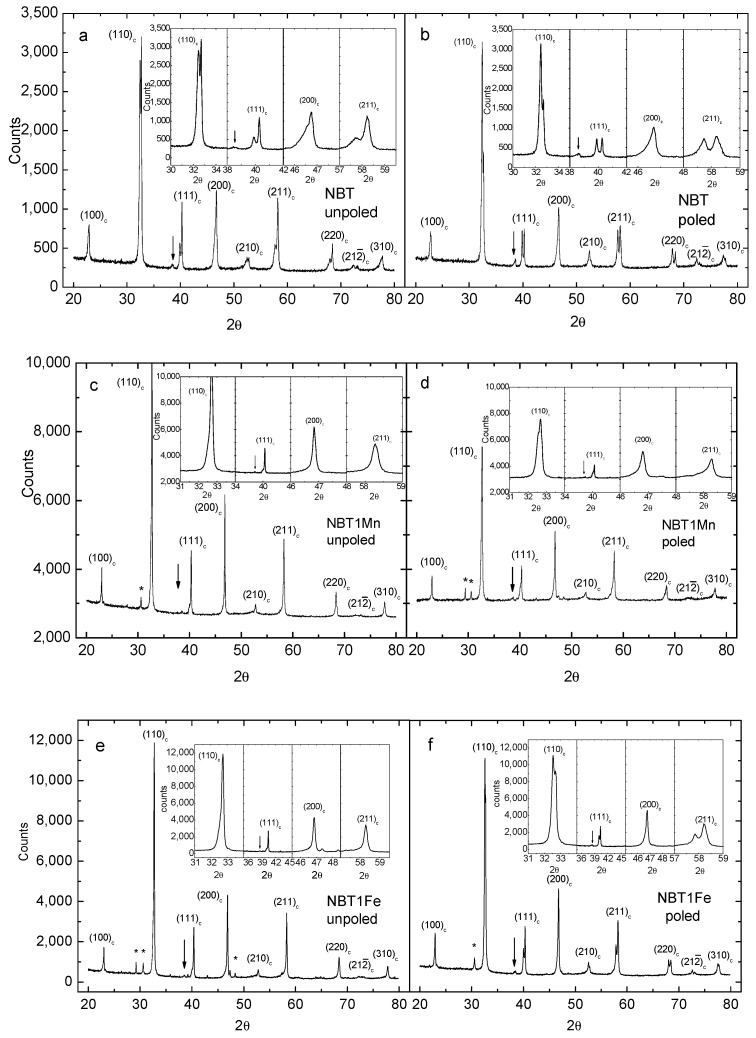
Room-temperature X-ray patterns of NBT (**a**,**b**), NBT1Mn (**c**,**d**) and NBT1Fe (**e**,**f**) ceramics in unpoled and poled states. The “c” subscript corresponds to the cubic phase. “*” shows the secondary phase. The arrow shows the super-lattice reflection of the rhombohedral R3c structure.

Figure 3 shows the room-temperature X-ray pattern of the NBT, NBT1Mn and NBT1Fe ceramics. As can be seen, all samples have a perovskite-type structure with rhombohedral symmetry. The phase of NBT is pure, whereas a small amount of a secondary Aurvillius phase is visible for both the Mn- and Fe-doped NBT (see also [19]).

**Figure 4:**
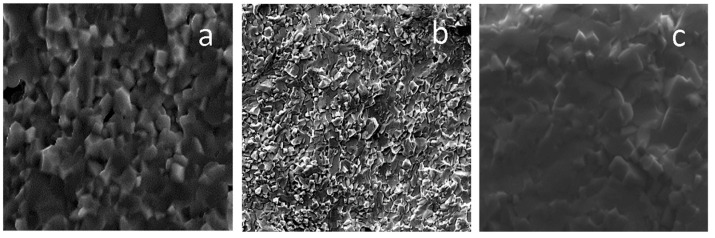
SEM micrographs of NBT (**a**), NBT1Mn (**b**) and NBT1Fe (**c**) ceramics.

SEM micrographs of the NBT, NBT1Mn and NBT1Fe ceramics are shown in Figure 4. While the pure NBT shows a unimodal grain size distribution with a grain size of ~6 μm, the microstructures of the Mn- and Fe-doped NBT revealed a bimodal grain size distribution consisting of grains with sizes of ~3 and ~1.5 μm (see also [19]).

**Figure 5:**
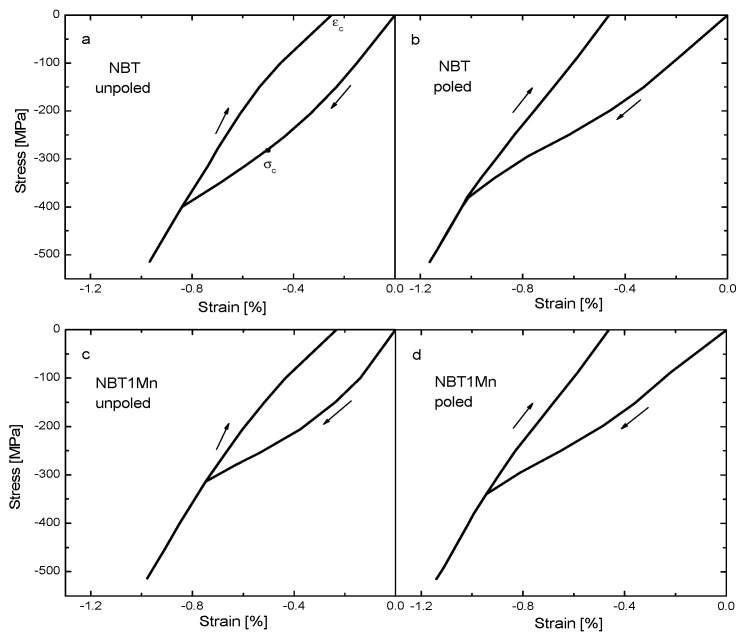
Stress–strain behaviors of NBT (**a**,**b**) and NBT1Mn (**c**,**d**) ceramics. The arrows illustrate the loading directions.

The stress–strain loops of NBT and NBT1Mn in the unpoled and poled states are shown in Figure 5. An initial linear behavior regime following Hooke’s law was observed, indicating a lack of ferroelastic domain reorientation. The appearance of coercive stress (indicated by the inflection point in the loading curve), the onset of strain saturation at higher stress levels and the development of a remnant strain (the strain at zero stress) upon unloading are clearly visible (see also Table 1).

After the linear regime in the stress–strain dependence, a nonlinear interval appeared, which was due to the development of spontaneous strain and alignment of the polarization direction perpendicular to the loading direction. With further increasing of the load, the domain structure saturated and the stress–strain dependence again followed Hooke’s law, where both reversible and irreversible domain switching can occur. However, during unloading, some domains switched back, reducing strain. Note that domain switching occurs over a wide range of applied stress and is most active at around 300 MPa for pure NBT and at around 240 MPa for Mn-doped NBT in the unpoled state. In contrast, in the PZT domain, switching occurs in a narrow interval of applied stress [22,23]. For the poled samples, the coercive stress decreased due to the residual stresses caused by the action of the electric field and the remnant strain increased due to non-180° domain switching (70 and 109° domain switching for our materials, which had rhombohedral symmetry) aligned parallel to the loading direction in the poling process. Young’s modulus decreased by about 15% at maximum load for all measured materials.

**Figure 6:**
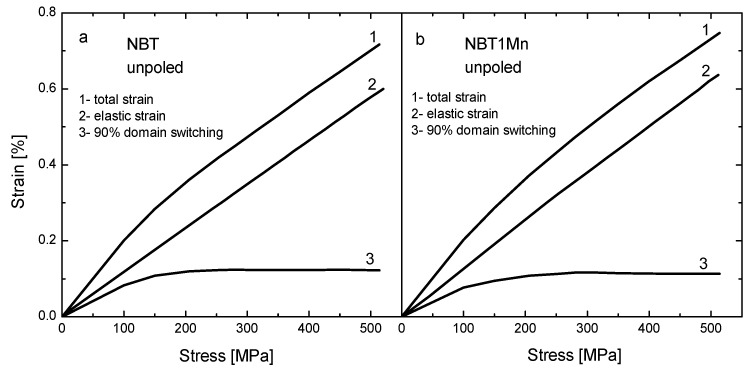
Stress–strain behavior measured in loading regime for as-received NBT (**a**) and NBT1Mn (**b**) ceramics.

The stress–strain behaviors measured for the as-received samples in the loading regime for the NBT and NBT1Mn ceramics are shown in Figure 6. The curves in this figure are the result of superimposing the stress-induced non-180° domain-switching strain with the elastic displacement strain [24]. To obtain the stress–strain curves corresponding only to domain switching, the elastic contribution should be eliminated by simply subtracting the straight lines, as shown in Figure 2. Although 180° domain rotation causes a change in polarization, no change in shape and very small atomic displacement occur; thus, there is no contribution to strain. For pure NBT, at stresses below about 180 MPa, only small switches occur; this extends up to 400 MPa with coercive stress of ~325 MPa. Exhaustion of domain switching appears at lower stresses, and coercive stress decreases as a result of both the introduction of additives to NBT and the action of the electric field (Figure 6 and Table 1). This is favorable for the application of these materials as a component of actuators.

**Figure 7:**
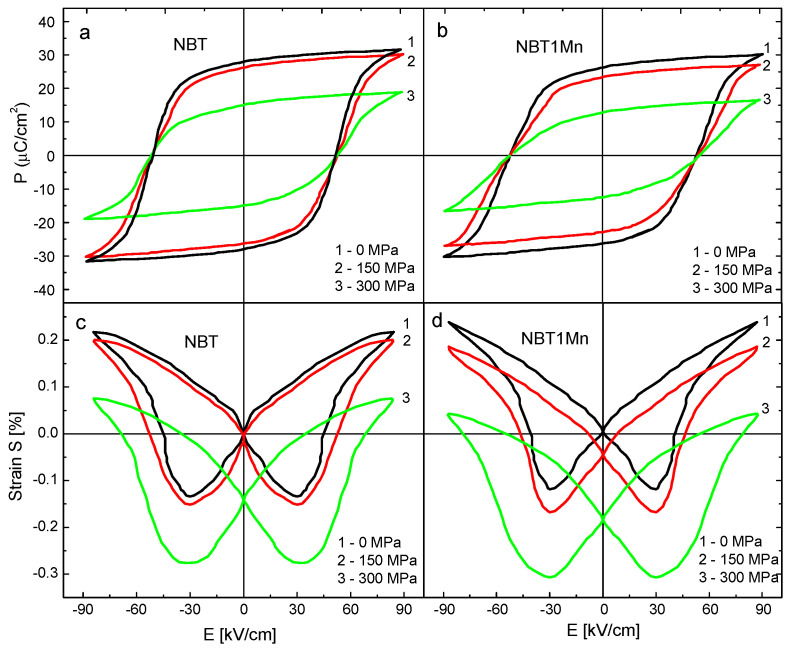
Polarization–electric field (P-E) and strain–electric field (S-E) hysteresis loops for NBT (**a**,**c**) and NBT1Mn (**b**,**d**) ceramics.

The hysteresis loops for polarization versus electric field P-E without compressive stress and under different compressive stresses, as well as strain versus electric field S-E without compressive stress and under different compressive stresses, of the NBT and NBT1Mn ceramics are shown in Figure 7. At S = 0, the square P-E and butterfly-shaped S-E curves clearly confirm the typical ferroelectric state of the ceramics. However, at a maximum field of 85 kV/cm, a larger strain was observed in the NBT with additives compared with the pure NBT. Both the maximum polarization and remnant polarization were significantly suppressed by the compressive stress, while the coercive field decreased slightly. However, a well- shaped P-E hysteresis loop persisted even at 300 MPa. The decrease in polarization should be attributed to the depolarization process induced by the compressive stress through non-180° ferroelastic domain switching, where the domains were aligned perpendicularly to the applied electric field. In other words, with increasing mechanical load, more and more domains are constrained by stress and cannot be reoriented by the electric field to participate in polarization reversal.

The area of the P-E hysteresis loop represents the unit-volume polarization dissipation energy of the material subjected to one full cycle of electric field loading and is related directly to the number of domains participating in the switching process in an electric field loading cycle. The area under the hysteresis loop is also used for estimation of the energy storage density and energy storage efficiency [25,26]. Progressive thinning, i.e., reduction in the P-E loop area, with increasing stress was observed (Figure 7), which indicated decreases in the dissipation energy and the number of domains contributing to polarization reversal and increases in both the energy storage density and energy storage efficiency. The decrease in the area under the hysteresis loop was more pronounced for the Mn-doped NBT in comparison with the pure NBT. These results can be utilized to further optimize the energy storage parameters for NBT and NBT-doped materials by the application of compressive stress.

With increasing stress levels, the shapes of the S-E curves changed (they became rounded) and were shifted toward negative strain. As mentioned, compressive stress induces elastic deformation and ferroelastic non-180° domain switching. Elastic deformation will shift strain curves downward; however, stress-induced ferroelastic domain switching can change the shapes of the S-E hysteresis loops.

**Figure 8:**
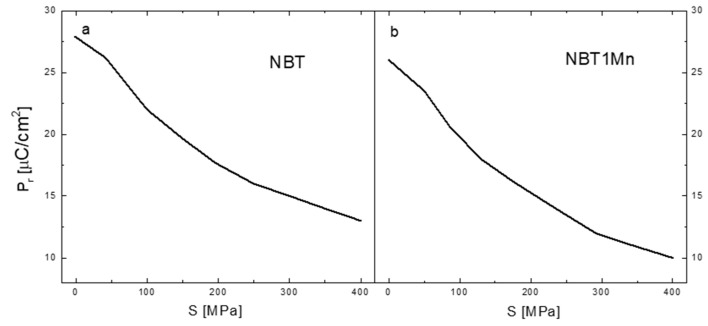
The change in the remnant polarization, P_r_, as a function of compressive stress for NBT (**a**) and NBT1Mn (**b**) ceramics.

The changes in the remnant polarization, P_r_, as a function of compressive stress for pure NBT and NBT1Mn are shown in Figure 8. Significant non-linear reduction in P_r_ with increasing mechanical stress can be observed for both ceramics. This decrease in the P_r_ was due to the combined effect of ferroelectric and ferroelastic domain switching, as mentioned above. The decrease in the P_r_ in the NBT1Mn is more pronounced than in the pure NBT, which means that NBT1Mn is more sensitive to mechanical stress than pure NBT. However, even at a stress of 400 MPa, the remnant polarization is still relatively high for both materials (~13.5 and ~10 μC/cm^2^ for pure NBT and NBT1Mn, respectively).

The results obtained for the remaining materials were very similar to those shown in Figure 3, Figure 4, Figure 5, Figure 6, Figure 7 and Figure 8 for Mn-doped NBT and did not provide any further essential information; therefore, for conciseness, they are not presented in this work. However, their characteristic ferroelastic parameters are listed in Table 1.

Pure NBT has two advantages for actuator applications; the first is high resistance to ferroelastic domain switching, and the second is large electric field-induced strain. However, actuator-pushing stresses for NBT that are higher than the coercive stress of 325 MPa can cause separation of the grain boundary interfaces and non-180° irreversible domain switching/rotation and can result in fatigue damage during operation as an actuator component. As coercive stress decreases for Mn- and Fe-doped NBT (Table 1), these issues will be minimized. Although both resistance to ferroelastic switching and electricity-induced strain decreased for these ceramics, they still had high values (much higher than hard PZT ceramics [22,23]). Therefore, these materials can be safely operated at sufficiently higher stresses. The enhanced actuation performance of Mn- and Fe-doped NBT must be addressed in the design and practical application of active actuators.

The introduction of additional ions causes a change in the stress values occurring in the material. The resulting stresses were calculated with the following formula:

s = Eη_d_ (1)

where E—Young’s modulus and η_d_—the deformation resulting from the introduction of the admixture. The latter was determined with the following formula:

η_d_ = (R_d_ − R_m_)/R_m_(R_m_/r)^3^ (2)

where R_d_—the radius of the introduced ion, R_m_—the radius of the native ion and r—the distance at which the deformation caused by the introduction of the ion was counted (1 nm). The deformations resulting from the introduction of Mn and Fe ions are illustrated in Table 2. The stresses arising after the introduction of iron and manganese ions into the NBT, presented in Table 2, are an order of magnitude smaller than the stresses arising in pure NBT during phase transformation [27]. This means that the introduction of these admixtures to NBT will not result in a change in symmetry. This is consistent with X-ray diffraction and Raman scattering results [19]. However, these admixtures have the effect of disturbing the structure on a local scale (variations in short-range crystalline order arise), which may result in improved macroscopic mechanical properties. Note that already-pure NBT shows structural disorder due to differences in the sizes and oxidation states of the monovalent Na^+^ and trivalent Bi^3+^ ions and 6 s^2^ Bi lone pair electrons at the A-site of the ABO_3_ perovskite structure. The incorporation of multivalent Mn and Fe ions introduces additional disorder in NBT. It is expected that both Mn and Fe ions can replace Ti cations.

The Vickers hardness (H_V_) was calculated using the following equation [28,29]:

H_V_ = 1.854P/d^2^ (3)

where P is load and d is the half-length of the long diagonal. The pure NBT has a high hardness of H_V_ 520 GPa, which increases after the introduction of Mn and Fe ions (Table 3). The hardness improvement in the Mn- and Fe-modified NBT was most likely caused by the effect of reduced grain size [18,30]. This hardness increases further after E-poling (Table 3), which can be related to the observed increase in remnant strain with poling (Table 1). The relatively high hardnesses of the investigated materials indicate the high strengths of their chemical bonds.

The critical stress intensity factor (K_Ic_) was calculated using the following formula [31]:

K_Ic_ = 0.016(E/H_V_)^0.5^(P/c^3/2^ (4)

where *E* is Young’s modulus, *P* is load and *c* is crack length. The critical stress intensity factor, K_Ic_, of the pure NBT is equal to 1.15 MPam^0.5^ (Table 3) and is close to that for PZT [7]. In comparison to other lead-free ferroelectrics like (Na_0.48_K_0.48_Li_0.4_)NbO_3_ (K_Ic_ = 0.48 MPam^0.5^) and (Na_0.5_K_0.5_)(Nb_0.9_Ta_0.1_)O_3_ (K_Ic_ = 0.8 MPam^0.5^) [32], the critical stress intensity factors of pure and Mn- and Fe-doped NBT are much higher. This factor increases further after E-poling (Table 3), which could be related to the observed increases in remnant strain for the poled samples. The increases in the critical stress intensity (fracture toughness) after both Mn and Fe ion introduction to the NBT and E-poling are consistent with the same trends of hardness and compressibility modulus changes (Table 3 and Table 4). Qualitatively, the increase in the fracture toughness is also in accordance with the increased bending strength (Table 5). The change in the K_Ic_ value could be associated with the influence of dopants both on the nature of the phase present and the microstructural features, i.e., grain and pore size and distribution. Although the introduction of dopants did not change the average crystal structure (symmetry), distortion of the local crystal structure and a small amount of Aurvillius phase appeared (see also [19]), also affecting the resistance of fractures. While the pure NBT showed a unimodal grain size distribution with a grain size of ~6 μm, the microstructures of the Mn- and Fe-doped NBT revealed a bimodal grain size distribution consisting of grains with sizes of ~3 and ~1.5 μm (see also [7]). Therefore, the higher K_Ic_ values for the NBT modified with Mn and Fe could also be attributed to their smaller grain size and bimodal grain size distribution.

An analysis of the experimental data presented in Table 2, Table 3, Table 4 and Table 5 showed that the introduction of Mn and Fe ions into NBT causes increases in the values of Young’s modulus (E), Poisson’s number (υ), the compressibility modulus (K), material hardness (H_V_), the critical stress intensity factor (K_Ic_) and bending strength (σ_m)_ and a slight decrease in the elastic modulus (G). Young’s modulus determines the stiffness of a material, the elastic modulus determines its susceptibility to plastic deformation and the compressibility modulus determines its fracture toughness. It is worth emphasizing that the bending strength is two-to-three times larger than that of conventional PZT ceramics.

The fact that the Young modulus had a higher value than the compressibility modulus indicates that the tested materials are difficult to break. This conclusion is confirmed by the very high bending strength of the tested materials: ~200 MPa (Table 5). The G/K ratio is a criterion for the brittleness/ductility of a material; if this ratio is less than 0.571, the material is ductile, and if it is greater, the material is brittle. Taking this criterion into account, the tested materials should be classified as ductile. Poisson’s number allows the estimation of types of chemical bonds. If its value is less than 0.1, there are covalent bonds in a material, while for ionic bonds, this value oscillates at around 0.25. The Poisson number values obtained for the tested materials indicate the ionic nature of their chemical bonds. It should be emphasized here that the Poisson number does not provide complete information about the nature of chemical bonds.

Ferroelectrics with perovskite structures contain ionic or ionic–covalent bonds. The crystal lattices of such materials offer strong resistance to moving dislocations, which is why they have relatively high hardnesses. E-poling of the tested materials causes enhancement of their mechanical parameters (Table 3, Table 4 and Table 5). This could be due to partially reconstructed structural disorder due to the action of the electric field. It should be noted that E-poling is a suitable, easy and fast procedure to enhance the mechanical behavior of the investigated materials for practical applications.

For isotropic ceramic materials, Young’s modulus (E), Kirchhoff’s modulus (G) and the compressibility modulus (K) satisfy the following relations [33,34]:

G/E ≈ 3/8 and K/G ≈ 5/3 (5)

The analysis of the obtained experimental data confirmed that the tested samples were characterized by a low anisotropy of approximately 1% and the above-mentioned relations were met, assuming, in the first case, a value of approximately 3.75 and, in the second case, of approximately 1.67. The high values of the parameters E, G and K of the tested materials allow them to be classified as so-called stiff materials.

Basically, the change in the mechanical properties of NBT after the introduction of Fe and Mn ions is influenced by two factors: (1) the decrease in the volume of the unit cell (according to the results of X-ray studies [19]) and (2) an increase in the covalence of the material (the electronegativity values of Mn, Fe and Ti are 1.55, 1.83 and 1.54 respectively). The decrease in the volume of the unit cell will shorten the chemical bonds and, consequently, strengthen the mechanical properties. The effect of the second factor will also strengthen the mechanical properties. As mentioned above, the increase in structural disorder with doping will also make contributions to the observed changes in these properties.

In general, the values of the piezoelectric coefficients increased after doping the NBT with the Mn and Fe ions (expected NBT1Fe, Table 6). This may be related to the distortion of the crystal lattice-generating stresses, which favor the formation of heterogeneous polar regions and additional interfacial energy, as well as local polarization perturbations facilitating domain reorientation [35,36]. However, the elastic compliances evaluated independently from the resonance and antiresonance frequencies decreased for the Mn- and Fe-doped NBT in comparison with the pure NBT (Table 6), which indicates that modified NBT ceramics become less deformed in response to applied force. This is in accordance with the higher hardness of these ceramics compared to pure NBT (Table 3). Note that the elastic compliances of the NBT and Mn- and Fe-doped NBT are almost half of those of the commercial PZT-based ceramics. It can be concluded that, considering the values of the piezoelectric parameters of the tested materials and their mechanical properties, they can be used in piezoelectric elements such as volumetric (bulk) acoustic wave resonators, electromechanical transducers and actuators [37].

The effective piezoelectric coefficients, d^∗^_33_, were also calculated from the strain–electric field (S-E) curves (Figure 7) according to the following formula [38]:

d^∗^_33_ = S_max_/E_max_ (6)

where S_max_ and E_max_ are the maximum strain and the maximum intensity of the electric field, respectively. The obtained values were 285 and 319 pm/V for the pure NBT and NBT1Mn, respectively.

## 4. Conclusions

In summary, the elastic modulus, fracture toughness and piezoelectric parameters of NBT and Mn/Fe-doped NBT ceramics in both the unpoled and poled states were examined for the first time. The results show that both doping and E-poling enhance the mechanical properties of these materials. This was explained to be mainly due to the nature of the phase present, the microstructural features, an increase in the covalence of the materials, stress on local scales and partially reconstructed structural disorder due to the action of the electric field. The elastic compliances of the investigated materials are far smaller than those of PZT-based ceramics, which indicates higher deformation resistance in response to applied force. In conjunction with the macroscopic stress–strain behavior, it was suggested that the investigated materials can be used in actuators where high blocking stress is desired. The presented properties compare favorably with those of other lead-free alternatives and are competitive with those of PZT, making Mn/Fe-doped NBT promising piezoelectric ceramics from a mechanical perspective. The presented findings indicate an effective and sustainable strategy for developing high-performance, lead-free piezoelectric materials.

## Figures and Tables

**Table 1:** Remnant strain (ε_r_) and coercive stress (σ_c_) of NBT and NBT doped with Mn and Fe ions for unpoled and poled samples.

Material	ε_r_ [%] (Unpoled/Poled)	σ_c_ [MPa] (Unpoled/Poled)
NBT	−0.27/−0.48	−325/−255
NBT05Mn	−0.28/−0.36	−287/−208
NBT1Mn	−0.29/−0.38	−286/−206
NBT05Fe	−0.28/−0.37	−285/−205
NBT1Fe	−0.28/−0.38	−283/−205

**Table 2:** Young’s modulus (E), deformation (η_d_) arising after the introduction of Mn and Fe ions into NBT and generated stresses (s) (Formulas (1) and (2)).

	E [GPa]	η_d_*10^−4^	s [GPa]
Material			
NBTMn	138		
Mn^2+^		0.8	0.011
Mn^3+^		0.1	0.001
Mn^4+^		0.3	0.004
NBTFe	137		
Fe^2+^		0.6	0.008
Fe^3+^		0.1	0.001
Fe^4+^		0.1	0.001

**Table 3:** Vickers hardness, H_V_ (GPa), and critical stress intensity factor, K_Ic_ (MPam^0.5^), for NBT and NBT doped with Mn and Fe ions for unpoled and poled samples.

	Hardness HV [GPa]	Critical Stress Intensity Factor
Material	(Unpoled/Poled)	K_Ic_ [MPam^0.5^] (Unpoled/Poled)
NBT	520/525	1.15/1.16
NBT05Mn	535/539	1.17/1.18
NBT1Mn	533/537	1.17/1.16
NBT05Fe	530/532	1.19/1.20
NBT1Fe	528/529	1.18/1.18

**Table 4:** Young’s modulus, E (GPa); elastic modulus, G (GPa); Poisson’s number, μ; compressibility modulus, K (GPa); and G/K ratio for NBT and NBT doped with Mn and Fe ions for unpoled and poled samples.

	E [GPa]	G [GPa]	μ	K [GPa]	G/K [GPa]
Material	(Unpoled/Poled)	(Unpoled/Poled)	(Unpoled/Poled)	(Unpoled/Poled)	(Unpoled/Poled)
NBT	136.95/137.57	52.87/53.03	0.295/0.297	92.91/92.15	0.569/0.575
NBT05Mn	138.02/138.08	52.09/51.85	0.347/0.352	92.94/92.96	0.561/0.558
NBT1Mn	137.93/137.99	50.71/50.64	0.324/0.331	92.84/92.97	0.546/0.545
NBT05Fe	137.52/137.54	50.65/50.54	0.382/0.389	92.93/92.61	0.545/0.546
NBT1Fe	137.16/137/53	51.23/51.87	0.354/0.361	92.52/92.61	0.554/0.560

**Table 5:** Bending strength, σ_m_ (MPa), for NBT and NBT doped with Mn and Fe ions for unpoled and poled samples.

	Bending Strength σ_m_ (MPa)
Material	(Unpoled/Poled)
NBT	200/205
NBT05Mn	203/205
NBT1Mn	204/207
NBT05Fe	202/204
NBT1Fe	203/205

**Table 6:** Piezoelectric parameters and elastic compliances of NBT and Mn- and Fe-doped NBT.

Material	$d_{33}[pC/N]$	$k_{33}[\%]$	$S_{11}^{E}[10^{-12}m^{2}/N]$	$S_{33}^{E}[10^{-12}m^{2}/N]$	$S_{44}^{E}[10^{-12}m^{2}/N]$
NBT	73	21	8.0	8.9	19.9
NBT05Mn	89	24	7.9	8.7	19.7
NBT1Mn	97	27	7.8	8.6	19.6
NBT05Fe	74	21	7.8	8.7	19.6
NBT1Fe	71	20	7.7	8.7	19.5

## Data Availability

The original contributions presented in the study are included in the article, further inquiries can be directed to the corresponding authors.
